# Health benefits of viewing nature through windows: A meta-analysis

**DOI:** 10.1093/biosci/biaf089

**Published:** 2025-07-04

**Authors:** Masashi Soga, Kevin J Gaston

**Affiliations:** Graduate School of Agricultural and Life Sciences, University of Tokyo, Tokyo, Japan; Environment and Sustainability Institute, University of Exeter, Penryn, England, United Kingdom

**Keywords:** ecosystem services, human–nature interactions, personalized ecology, nature dose, urbanization

## Abstract

Experiencing nature offers numerous health and well-being benefits, particularly for urban residents. Although the benefits of visiting natural environments are well documented, less is known about the health effects of experiencing nature without going outdoors—in particular, viewing it through building windows. This meta-analysis synthesizes findings from 28 studies encompassing 104 results to examine the relationship between window views of nature and human health. Improvements were reported across various physiological, psychological, and physical health measures, with most studies focused on psychological outcomes. The meta-analytic results indicate consistently positive effects, with particularly strong benefits in studies using physiological health measures and focusing on nature in urban settings. Although some publication bias was detected, correcting for it did not change the overall conclusions. These findings highlight the potential of integrating nature views into built environments as a practical strategy for enhancing public health, particularly in urban areas.

Although urbanization offers many benefits, including improved access to healthcare, it is also increasingly recognized as one of the most significant global health challenges of the twenty-first century. Indeed, urban living is often associated with a high prevalence of chronic and noncommunicable physical and psychological health conditions (Moore et al. 2003, Dye 2008, Peen et al. 2010). There is growing recognition of the important role that nature experiences can play in addressing this issue, supported by substantial evidence demonstrating their association with a wide range of positive health outcomes (Keniger et al. 2013, Hartig et al. 2014, Bratman et al. 2019, Marselle et al. 2021). These include improvements in physical health (e.g., reduced blood pressure and decreased mortality from cardiovascular diseases), psychological health (e.g., lower stress levels and enhanced cognitive function), and social well-being (Keniger et al. 2013, Hartig et al. 2014). Consequently, particularly in higher-income nations, there is an increasing emphasis on fostering direct interactions with nature through initiatives such as nature prescriptions (Kondo et al. 2020) and nature-based health interventions (Shanahan et al. 2019).

So far, both scientific and policy discussions about the health benefits of nature experiences have primarily focused on the advantages of physically being in natural environments, such as visiting green spaces (Bratman et al. 2012, Twohig-Bennett and Jones 2018, Coventry et al. 2021). However, people also experience nature without being physically present by engaging their senses—sight, sound, and smell (Gaston and Soga 2020, Gaston 2024). One of the most common forms of such sensory engagement is viewing nature through building windows (Kaplan 1993, Cox et al. 2017, Soga et al. 2021). In a now-classic study, Ulrich (1984) demonstrated that postsurgery patients with hospital windows overlooking trees rather than a brick wall recovered more quickly and required less pain medication. More recent research has shown that viewing nature through windows is associated with various health benefits, including reduced symptoms of depression and anxiety (Soga et al. 2021), increased life satisfaction (Chang et al. 2020), improved cognitive function (Li and Sullivan 2016), and decreased loneliness (Soga et al. 2021). However, no formal evidence synthesis of these findings has been conducted to date, leaving it unclear how consistent these positive effects are across studies and the extent of the benefits such experiences can provide.

Better understanding of the role of viewing nature through windows in promoting health could enhance efforts more fully to use urban nature for improving well-being. In many urbanized and developed societies, people spend most of their time indoors, whether at home or work (Klepeis et al. 2001, Leech et al. 2002). As such, window views of nature likely represent a significant and perhaps the larger portion of the nature experiences people encounter in their daily lives (Cox et al. 2017). Moreover, this form of nature engagement is accessible to individuals with limited opportunities to visit natural environments (e.g., those living in areas with scarce greenspace or who have little time for outdoor recreational activities), limited motivation (e.g., those concerned about safety issues related to outdoor activities), or limited capability (e.g., older adults or individuals with disabilities). Incorporating views of nature into health strategies—such as by planting trees or creating pocket parks near homes and workplaces—could therefore significantly expand the number of people who can benefit from nature experiences (Wang et al. 2021).

In the present article, we present the results of a systematic review and meta-analysis of published studies examining the association between viewing nature through windows and human health. For this study, we define viewing nature through windows as the visual observation of all (nonhuman) living organisms and ecosystems—such as trees, mountains, lakes, and gardens—through building windows (Gaston and Soga 2020). Health is defined in accordance with the WHO's definition: a state of complete physical, mental, and social well-being and not merely the absence of disease or infirmity (WHO 1946). To conduct our meta-analysis, we systematically reviewed literature, including that of public health, psychology, architecture, and urban planning, and identified studies investigating the relationship between nature viewing and health outcomes. Using a standardized meta-analytic approach, we evaluated the overall association between nature viewing and health outcomes across the selected studies. We then examined how the strength of this relationship may vary on the basis of study design, participant characteristics, the type of nature viewed, and the health outcomes measured. Finally, on the basis of the findings from our review, we identify key knowledge gaps in this area and offer recommendations for future research.

## Reviewing studies on nature views and health

**Figure 1.**
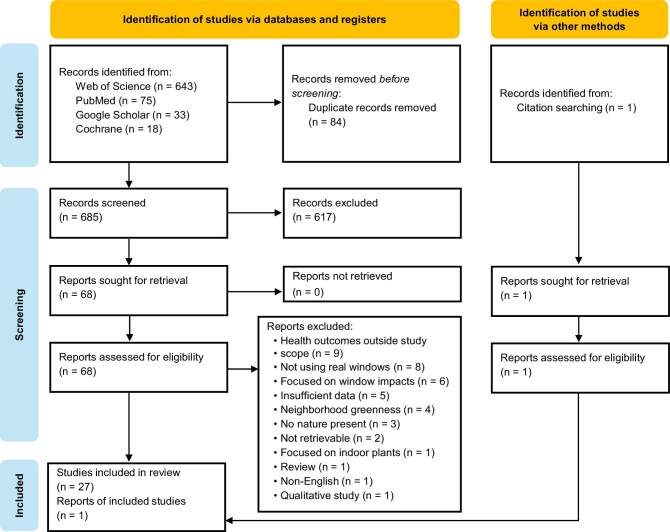
The process and outcome of the literature search. After we removed duplicates for the database search, 685 articles remained. During the title and abstract screening stage, we excluded studies that did not focus on the relationship between viewing nature through windows and health, as well as nonempirical studies. Following this, we reviewed the full text of 68 articles and identified 27 case studies. To identify additional relevant studies, we examined the reference lists of these 27 articles, which led to one more study being eligible for inclusion in the meta-analysis.

We conducted a systematic literature search in accordance with the Preferred Reporting Items for Systematic Reviews and Meta-Analyses guidelines (Page et al. 2021). On 24 December 2024, we searched four research article databases—the ISI Web of Science, PubMed, Cochrane, and Google Scholar—using keyword strings related to window views and health. To avoid missing key studies, we employed a broad range of search terms related to both window views and health outcomes (see supplemental appendix S1). Studies were included if they met the following criteria: They investigated the relationship between viewing nature through windows and health, they employed a quantitative approach, and they provided statistical methods or sufficient statistical information relevant to this relationship. We excluded studies that investigated window views in virtual environments (e.g., VR or photos) rather than real physical windows (e.g., Jing et al. 2024), that assessed the health benefits of window presence without specifically focusing on nature views (e.g., Ko et al. 2020), or that examined perceptions and attitudes not directly related to health, such as salary satisfaction, neighborhood satisfaction, perceptions of room brightness, or room satisfaction (e.g., Lottrup et al. 2015). The search was limited to English-language articles, with no restrictions on publication year, and grey literature was excluded. The database search initially identified 27 relevant studies (see figure 1 for the search process and outcomes). In addition, we reviewed the reference lists of these selected studies and identified one more relevant study, resulting in a final total of 28 case studies.

For each identified study, we recorded the following characteristics: the author's name, the year of publication, the study design, the study location (country or region), the study context, the type of nature viewed, the number and characteristics of samples, the health outcomes measured, and the results. The study designs, study context, type of participants, type of nature viewed, and health outcomes were categorized as follows (see supplemental appendix S2 for more details on the classification strategy): the study design (interventional or noninterventional), the study context (residential, workplace, educational, healthcare, leisure, and correctional), the type of participants (female biased or non–female biased), the type of nature viewed (wild, managed, and general), and the health outcomes (physiological, psychological, and physical).

For the results, we extracted statistics necessary for conducting a meta-analysis (see the “Synthesizing evidence” section). In cases where statistics were presented as figures rather than raw data, we used PlotDigitizer software to extract the data. If a single study reported results for more than one health outcome, we treated each result (hereafter referred to as a *study result*) as independent (see also the “Data analysis” section). To avoid pseudoreplication, we excluded duplicate results derived from repeated analyses, such as subgroup analyses. Study results that only presented significant findings, without including nonsignificant results, were removed to prevent overestimation of the effects. We assessed the quality of study design using the Mixed Methods Appraisal Tool (MMAT), which is designed to evaluate studies employing diverse methodological approaches (Hong et al. 2019; see supplemental appendix S3).

## Summary of the included studies

**Figure 2.**
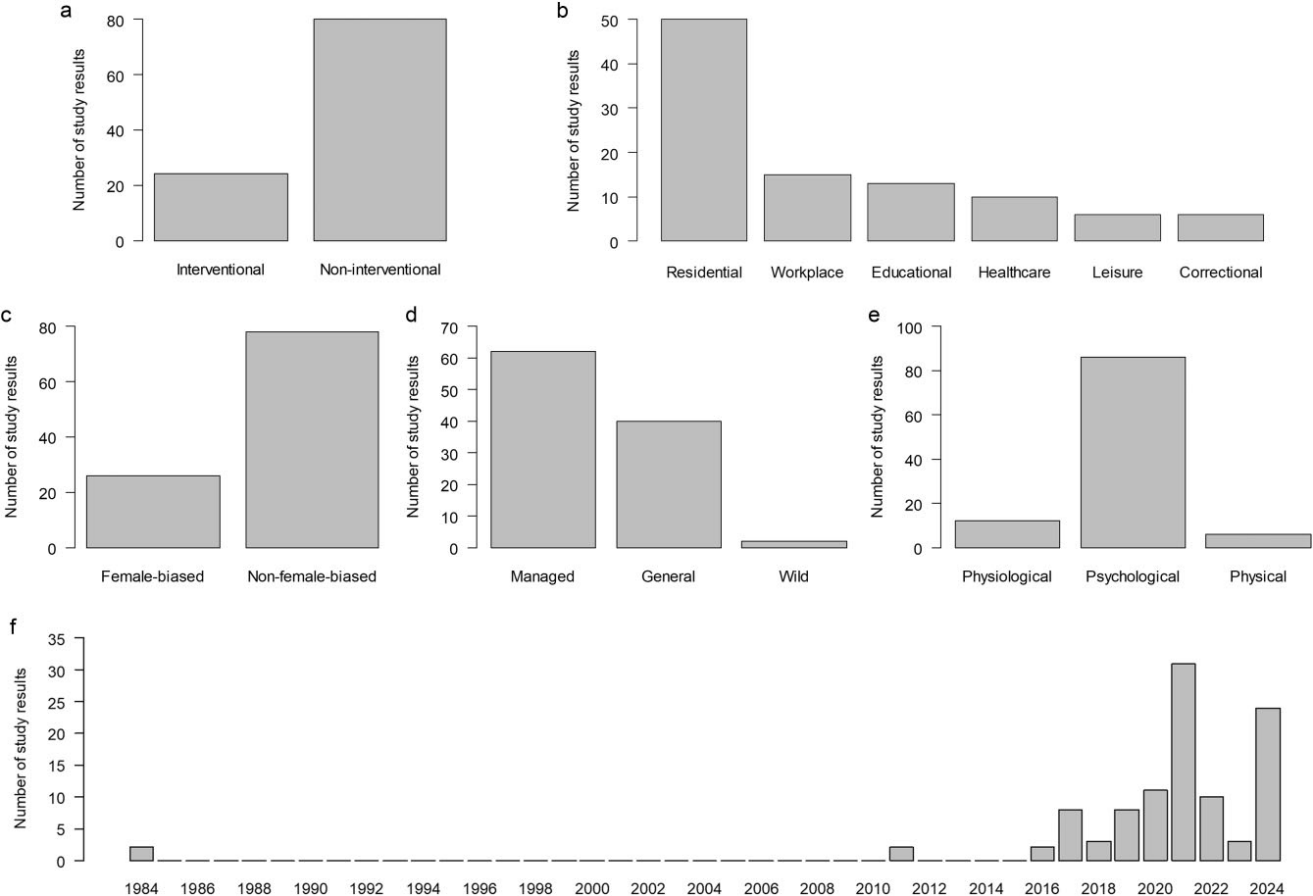
Overview of the characteristics of the 28 case studies (104 study results). The study results are categorized by (a) study design (interventional or noninterventional), (b) study context (residential, workplace, educational, healthcare, leisure, or correctional), (c) sample characteristics (female-biased or non-female-biased), (d) type of nature viewed (managed, general, or wild), (e) type of health outcomes (physiological, psychological, or physical), and (f) publication year.

The 28 included studies yielded a total of 104 independent study results (supplemental appendix S4). These study results varied widely in terms of study design, geographic location, study context, type of participants, type of nature examined, sample characteristics, and measured health outcomes (appendix S4). Most of the study results were derived from noninterventional studies (figure 2a). The studies investigated various contexts, with residential settings being the most common, followed by workplace, healthcare, educational, leisure, and correctional environments (figure 2b). A quarter of the study results were based on female-biased samples (figure 2c). Approximately 60% of the study results focused explicitly on nature in urban settings (managed nature), whereas only a small proportion addressed that in more wild settings (wild nature; figure 2d). Sample characteristics also varied considerably, with studies including participants from diverse demographic backgrounds, such as elementary schoolchildren, postpartum women, and male prisoners (appendix S4). In addition, around 80% of the study results examined psychological health outcomes, whereas many fewer focused on physiological or physical outcomes (figure 2e). Most of the included studies were published after 2015 (figure 2f), nearly 30 years after Ulrich (1984) first introduced the concept of the health benefits of viewing nature through windows. Notably, the majority of studies published after 2020 were conducted in the context of the COVID-19 pandemic. The majority of included studies (27 out of 28; 96%) met at least half of the MMAT quality criteria (appendix S3).

The 104 study results demonstrated a positive association between viewing nature through windows and various aspects of human health (appendix S4). The reported benefits included reduced symptoms of depression and anxiety (Emami et al. 2018, Braçe et al. 2020, Soga et al. 2021, Yen et al. 2024), decreased stress (Li and Sullivan 2016, Elsadek et al. 2020), and lower levels of negative emotions such as anger and confusion (Spano et al. 2021). Studies also documented improvements in positive emotions, including happiness (Soga et al. 2021) and life satisfaction (Chang et al. 2020, Soga et al. 2021), as well as enhanced cognitive function (Zhang et al. 2024). Additional benefits included faster postoperative recovery (Mascherek et al. 2022) and reduced pain perception following surgery (Emami et al. 2018). Collectively, these findings suggest that viewing nature through windows can contribute to a broad spectrum of health and well-being outcomes.

## Overall meta-analytic results

We synthesized the findings from the selected studies using a meta-analytic approach. As the methodological quality of most studies was acceptable (see appendix S3), no studies were excluded on this basis. To quantify the strength of the relationship between viewing nature through windows and health outcomes, we used Pearson's correlation coefficient (*r*). If studies reported statistics other than *r*, we converted them to *r* using the formulae provided in supplemental appendix S5. For the meta-analysis, we standardized correlation coefficients into *z*-scores using Fisher's *r*-to-*z* transformation, analyzed them, and then back transformed the results to *r* (Hedges and Olkin 1985). We assessed heterogeneity among study results using Cochran's *Q* test and the *I*² index statistic (Higgins et al. 2003). All analyses were conducted in R version 4.4.0 (R Core Team 2024) using the “metacor” function from the “meta” package (Schwarzer 2007).

**Figure 3.**
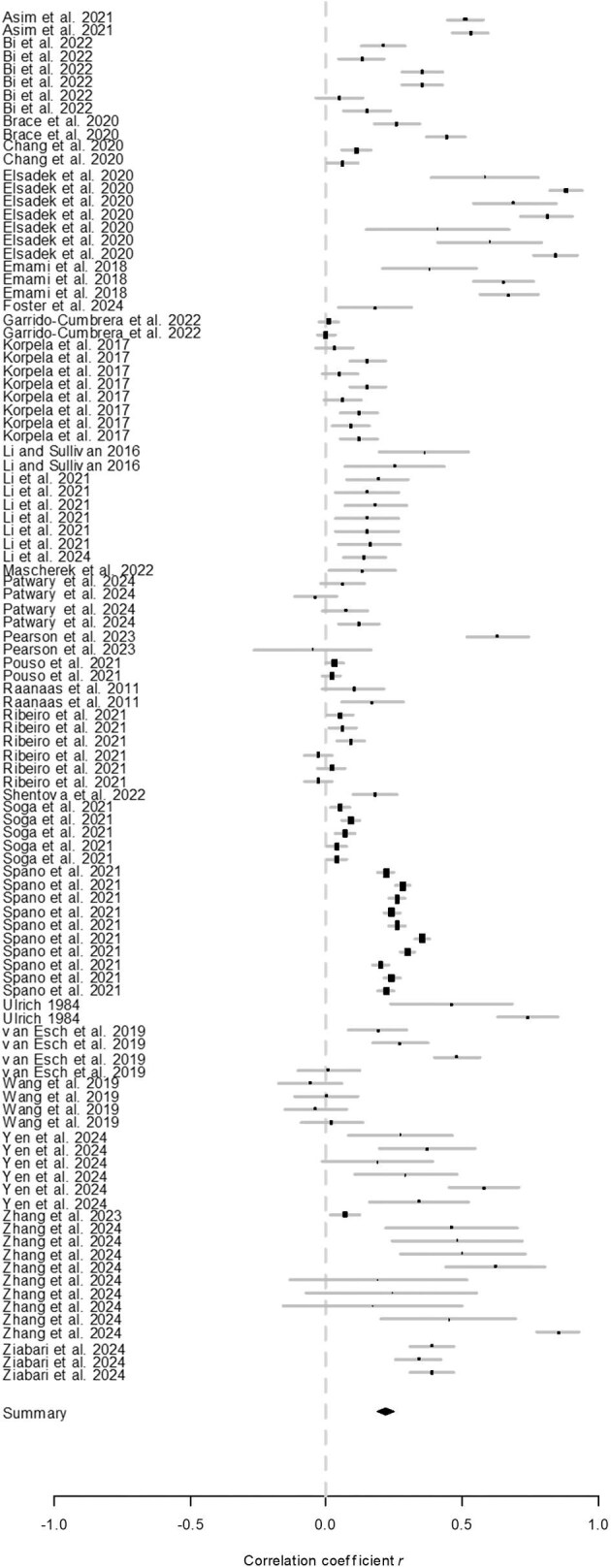
Results of overall meta-analysis of the relationship between viewing nature through windows and human health.

We first calculated the overall effect size estimates and 95% confidence intervals (CIs) for the association using a random-effects model. The analysis revealed a significant positive estimated mean correlation of *r* = .25 (95% CI = 0.20–0.29, *p* < .001; figure 3). Because many studies reported multiple results, there was a possibility of pseudoreplication affecting the outcome. To account for this, we recalculated the overall effect size by sampling only one result per study. Using 1000 bootstrap resamples in R, we estimated the mean and 95% CI of the effect size. This analysis confirmed that the overall effect size remained significantly positive (mean = 0.23; see supplemental appendix S6), suggesting that pseudoreplication is unlikely to have influenced the results.

## Variation in health responses to nature views across the literature

Across the 104 study results, there was substantial heterogeneity (*Q*(103) = 1965.68, *p* < .001; *I*² = 94.8%), indicating considerable variability in effect sizes. This suggests that multiple factors may moderate the strength of the relationship between viewing nature through windows and health. To explore these potential moderators, we tested whether pooled effect sizes varied by study design, participant type, nature type, and health outcome. We assessed the statistical significance of moderator variables using the Cochran's *Q* test.

The strength of the association between viewing nature through windows and health varied depending on the four factors we examined (table 1). The pooled effect size was more than twice as high in studies using an interventional design as it was in noninterventional studies (table 1). Two key factors may explain this difference. First, interventional studies are specifically designed to assess the health benefits of viewing nature, whereas noninterventional studies may be more susceptible to confounding influences. Second, interventional studies are often focused on health outcomes that can change within minutes or hours of exposure to a nature view, such as heart rate and mood disturbances (e.g., Elsadek et al. 2020, Zhang et al. 2024). Therefore, interventional studies are considered more likely clearly to detect health benefits of viewing nature through windows.

**Table 1.** Results of four moderator analyses.

					Heterogeneity			Between-subgroup difference		
Subgroup category	Subgroup	Number of study results	Effect size (mean)	95% confidence interval	*Q*	df	*I*²	*Q*	df	*p*
Study design	Interventional	24	0.47	0.32–0.59	248.27	23	90.7	11.09	1	.001
	Noninterventional	80	0.20	0.16–0.23	1716.17	79	95.4	–	–	–
Type of participants	Female biased	26	0.36	0.23–0.48	333.67	26		4.75	1	.03
	Non–female biased	78	0.21	0.17–0.25	1630.37	78		–	–	–
Type of health outcomes	Physiological	12	0.57	0.42–0.69	36.04	11	69.5	50.78	2	.001
	Psychological	86	0.22	0.18–0.26	1807.14	85	95.3	–	–	–
	Physical	6	0.02	–0.05–0.08	12.28	5	59.3	–	–	–
Type of nature	Managed	62	0.29	0.22–0.36	786.32	61	92.2	8.65	2	.01
	Wild	2	0.14	0.05–0.22	0.7	1	0	–	–	–
	General	40	0.20	0.14–0.25	1143.28	39	96.6	–	–	–

*Note:* A meta-analysis was conducted across ten subgroups based on study design, type of participants, type of nature, and type of health outcomes. Heterogeneity of each subgroup and between-subgroup difference was assessed using Cochran's *Q* test.

The pooled effect size was higher in studies with female-biased samples (*r* = .36) than in those without a female bias (table 1). This is consistent with previous research showing that females tend to experience greater health benefits from nature than males (see Núñez et al. 2022). One possible explanation for this difference is that females generally have a stronger emotional connection to nature (Price et al. 2022, Rosa et al. 2023). Recent studies suggest that this emotional connection can enhance the psychological benefits of nature experiences (e.g., Colléony et al. 2020, Gu et al. 2023). Given this, females may be more likely to experience health benefits from viewing nature through windows.

Effect sizes also varied depending on the type of health outcome measured, with physiological outcomes showing the strongest effect, followed by psychological and physical outcomes (table 1). These differences may be due to the different timescales over which these health indicators respond to nature exposure. Physiological responses, such as changes in skin conductance or heart rate, typically occur within minutes to hours of viewing nature. In contrast, psychological and physical health outcomes—such as reductions in depressive symptoms, improvements in life satisfaction, or postsurgery recovery—often take days to months to emerge. The longer timeframe required to assess these outcomes increases the likelihood that other environmental and social factors may influence the results.

The pooled effect size was higher for studies focused on managed nature than for those focused on nonmanaged (general and wild) nature (table 1). Several factors may explain this difference. First, managed nature—such as gardens and roadside trees—is often located closer to buildings where people live and work than are wild landscapes (e.g., forests or mountains). This makes it more likely to provide immediate experiences with nature (Gaston and Soga 2020). Second, managed environments are often intentionally designed for aesthetic appeal, whereas certain types of wild nature, such as dense forests or overgrown vegetation, may evoke feelings of fear or discomfort (Bixler and Floyd 1997, Andrews and Gatersleben 2010, Fischer et al. 2024). Although this study could not pinpoint the exact reasons for the differences in health benefits between managed and unmanaged nature, our findings emphasize the importance of urban greenery in enhancing health and well-being, particularly for urban populations.

Although the patterns identified in these moderator analyses are plausible and consistent with existing literature, some findings should be interpreted with caution. Our meta-analysis included 28 case studies, and several moderator categories were based on a limited number of study results. This was particularly true for the physical health outcome category (*n* = 6) and the wild nature category (*n* = 2), both of which showed relatively weak associations between nature views and health. Further research is needed more accurately to assess the magnitude of health benefits related to physical outcomes and views of wild nature.

Clearly, beyond the four moderators examined, other factors are also likely to influence the strength of the relationship between window views of nature and health. One such factor is the quantity and quality of nature views participants had. Although we could not include this aspect in the moderator analysis because of limited data on the frequency and duration of nature viewing in the selected studies (appendix S2), these factors likely influence the strength of the relationship. The socioeconomic status of the participants may also shape this association. For example, more affluent areas tend to support higher biodiversity—a phenomenon known as the *luxury effect* (Leong et al. 2018)—which may lead to greater health benefits from nature views. In addition, the participants’ stress levels at the time of the study may also affect the results. For example, studies conducted during COVID-19 lockdowns (e.g., Soga et al. 2021, Yen et al. 2024) or those focusing on office workers experiencing psychological distress (Elsadek et al. 2020) are more likely to detect positive impacts of nature views on health outcomes. A comprehensive understanding of the factors shaping these effects will offer insights into how, when, and where nature views should be provided to maximize their health benefits.

## Publication bias

**Figure 4.**
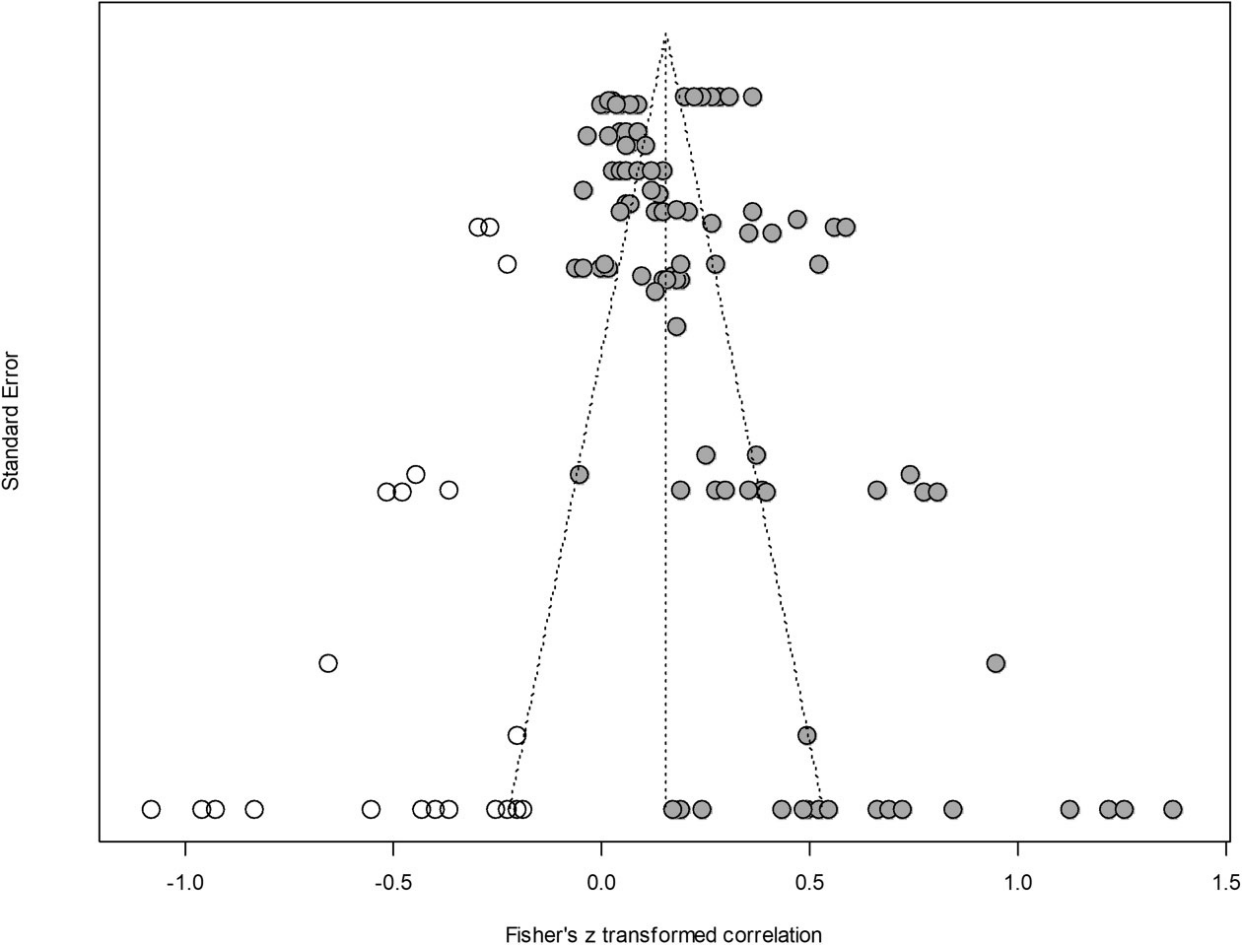
Assessment of potential publication bias through a trim-and-fill analysis (***n*** = 104). The filled circles represent the observed data (104 study results), with the ***x***-axis displaying the effect size measure (Fisher's ***z***-transformed correlation) and the ***y***-axis representing study precision (standard error). The trim-and-fill analysis, conducted using the R0 estimator (Duval and Tweedie 2000) via the “trimfill” function from the “metafor” package (Viechtbauer 2010) in R, identified 21 missing results, shown as open circles.

To assess potential publication bias, we constructed a funnel plot and performed Egger's regression test for funnel plot asymmetry (Egger et al. 1997). The test indicated the presence of publication bias (*p* = .01), and the funnel plot showed strong asymmetry among the 104 study results (the gray circles in figure 4). To account for this bias, we conducted a trim-and-fill analysis to estimate the number of missing studies and adjust the effect size accordingly. The analysis suggested that 21 study results were missing from the data set (the white circles in figure 4). After imputing the missing data, the association between viewing nature through windows and health remained significant but was attenuated (*r* = .15; 95% CI = 0.09–0.22).

## Research gaps in the literature

Through this systematic review, we identified several key areas where further research could enhance understanding of the health benefits of viewing nature through windows. One major gap is the limited understanding of the dose–response relationship between viewing nature through windows and health outcomes. In our systematic review, most studies examined these benefits by comparing individuals with and without nature views (e.g., van Esch et al. 2019, Elsadek et al. 2020, Zhang et al. 2024) or by assuming a linear relationship between nature exposure and health (e.g., Soga et al. 2021, Bi et al. 2022, Yen et al. 2024). Although such simplifications may be useful in early stage research, obtaining evidence that is practically more actionable requires a deeper understanding of the complexity of this relationship. For example, if the relationship between the greenness of window views and psychological health follows a hump-shaped pattern, there may be an optimal level of greenness that maximizes benefits (Shanahan et al. 2015). Indeed, research suggests that although having more vegetation near windows can enhance well-being, it can also block sunlight and, in some cases, the negative effects of reduced sunlight could outweigh the benefits of additional greenery (Mascherek et al. 2022).

Second, there is a need to clarify the relative importance of outdoor versus indoor nature experiences for human health. Most of the studies included in our review were focused solely on viewing nature through windows, with little consideration of how these benefits compare with those of outdoor nature experiences (e.g., Soga et al. 2021). As a result, it remains uncertain to what extent a decline in outdoor nature experiences can be compensated for by increased exposure to nature through windows and which aspects of health benefits are particularly advantageous in indoor settings. Although some benefits, particularly those related to physical activity, cannot be gained simply by viewing nature, window views may offer greater psychological benefits because, for most people, these indoor nature experiences occur much more frequently and for longer durations.

Third, it is important to know about which specific ecological and environmental features contribute most to the benefits of views from windows. Among the 28 case studies included in our review, most defined nature in broad terms, such as natural environments or greenery (e.g., Asim et al. 2021, Spano et al. 2021, Ziabari et al. 2023), whereas studies examining the effects of specific ecological attributes on health remain scarce (e.g., Pearson et al. 2023a). One possible reason for this gap is the limited involvement of environmental sciences in this field, because most existing research originates from psychology and public health. Greater engagement with ecology could help address this gap (Pearson et al. 2023b), because it offers sophisticated tools for quantifying biological characteristics of natural environments, such as habitat heterogeneity and vegetation structure.

Fourth, given the positive health impacts of nature views through windows, as we have demonstrated in this article, it is crucial to understand how these benefits are distributed among different people in society. Access to nature in both public and private spaces is often skewed toward individuals with higher incomes (Sharifi et al. 2021). Arguably, this pattern likely extends to nature views through windows, with wealthier individuals having access both to a greater quantity and higher quality of such views. In fact, residences with better nature views tend to have higher economic value (Jim and Chen 2006). Understanding the extent and nature of these disparities is essential for developing strategies to reduce health inequities and identifying areas where increasing access to nature views can have the greatest impact.

## Implications

On the basis of a meta-analysis of 104 study results, we found that viewing nature through a window is associated with a wide range of health benefits, particularly psychological outcomes, and the association was consistently positive. Even after adjusting for publication bias, the results remained significant, reinforcing the reliability and robustness of our conclusions. Although this positive association does not necessarily establish causation, following the Bradford Hill criteria (Hill 1965), a causal relationship seems plausible for several reasons. First, the pooled effect size was comparable to those reported in public health studies examining the relationship between other well-established environmental factors and human health (Rojas-Rueda et al. 2021). Second, the association was observed across diverse groups (e.g., employees, students, patients) and settings (e.g., residential, educational, workplace), demonstrating its consistency and generalizability. Third, several theoretical frameworks, including attention restoration theory (Kaplan and Kaplan 1989), stress reduction theory (Ulrich et al. 1991), and the greenery hypothesis (Fukano and Soga 2024), provide mechanistic explanations for the link between viewing nature and improved health. Fourth, moderator analyses consistently showed that interventional studies report positive health effects from viewing nature, strengthening the causal inference. To further strengthen causal inference and inform evidence-based practice, more interventional studies—particularly those employing long-term study designs—are needed directly to test the health effects of viewing nature through windows.

Our findings emphasize the value of integrating nature views into urban environments to enhance health and well-being, particularly in densely populated cities where access to large green spaces is limited. Achieving this will require collaboration among various stakeholders, including urban planners, architects, landscape designers, and public health professionals, and can be realized through multiple approaches, such as architectural design and urban planning. Although recent building designs often prioritize energy efficiency by reducing the window-to-wall ratio (Belussi et al. 2019), future designs should also focus on maximizing people's exposure to nature by carefully considering the placement and size of windows. In addition, strategically planting trees and creating gardens in locations visible from residential and workplace windows could further enhance people's nature experiences (Cox et al. 2019). By incorporating these design elements, urban environments could support well-being even for individuals with limited access to outdoor nature. In this sense, nature views through windows likely create windows of opportunity for healthier, more inclusive, and more sustainable city planning.

## Supplementary Material

biaf089_Supplemental_File

## Data and materials availability

All data used in this study are provided in the supplemental material.

## Author Biography

Masashi Soga (asoga@g.ecc.u-tokyo.ac.jp) is affiliated with the Graduate School of Agricultural and Life Sciences, at the University of Tokyo, in Tokyo, Japan. Kevin J. Gaston is affiliated with the Environment and Sustainability Institute, at the University of Exeter, in Penryn, England, in the United Kingdom.
