# Supplementary material for: Health benefits of viewing nature through windows: A meta-analysis
Source: Bioscience. 2025 Jul 4;75(8):628–36. doi: 10.1093/biosci/biaf089 (PMC12352305; doi:10.1093/biosci/biaf089)

**Appendix 1. Search terms used in this review.**

| Database | Search query |
| --- | --- |
| Web of Science | TS =(("nature view*" OR "natural view*" OR "green view*" OR "window view*" OR "nature window*" OR "natural window*" OR "view* of nature" OR "nature through window*" OR "nature of the view*") AND ("health" OR "wellbeing" OR "well-being" OR "depression*" OR "stress" OR “quality of life” OR “satisfaction” OR “happiness” OR “mood” OR "cognitive function*" OR "academic performance*" OR "brain" OR "emotion*" OR "psycholog*" OR "physiolog*" OR "recovery" OR “fatigue”)) |
| PubMed | (("nature view*" [Title/Abstract] OR "natural view*" [Title/Abstract] OR "green view*" [Title/Abstract] OR "window view*" [Title/Abstract] OR "nature window*" [Title/Abstract] OR "natural window*" [Title/Abstract] OR "view* of nature" [Title/Abstract] OR "nature through window*" [Title/Abstract] OR "nature of the view*" [Title/Abstract]) AND ("health" [Title/Abstract] OR "wellbeing" [Title/Abstract] OR "well-being" [Title/Abstract] OR "depression" [Title/Abstract] OR "stress" [Title/Abstract] OR “quality of life” [Title/Abstract] OR “satisfaction” [Title/Abstract] OR “happiness” [Title/Abstract] OR “mood” [Title/Abstract] OR "cognitive function*" [Title/Abstract] OR "academic performance*" [Title/Abstract] OR "brain" [Title/Abstract] OR "psycholog*" [Title/Abstract] OR "physiolog*"[Title/Abstract] OR "recovery"[Title/Abstract] OR "fatigue"[Title/Abstract])) |
| Cochrane | ("nature view" OR "nature views" OR "natural view" OR "natural views" OR "green view" OR "green views" OR "window view" OR "window views" OR "nature window" OR "nature windows" OR "view of nature" OR "views of nature" OR "nature through window" OR "nature through windows") AND ("health" OR "wellbeing" OR "well-being" OR "depression" OR "depressions" OR "stress" OR "quality of life" OR "satisfaction" OR "happiness" OR "mood" OR "cognitive function" OR "cognitive functions" OR "academic performance" OR "academic performances" OR "brain" OR "emotion" OR "emotions" OR "psychology" OR "physiology" OR "recovery" OR “fatigue”) |
| Google Scholar | allintitle: ("nature view*" OR "natural view*" OR "green view*" OR "window view*" OR "nature window*" OR "natural window*" OR "view* of nature" OR "nature through window*" OR "nature of the view*") ("health" OR "wellbeing" OR "well-being" OR "depression*" OR "stress" OR “quality of life” OR “satisfaction” OR “happiness” OR “mood” OR "cognitive function*" OR "academic performance*" OR "brain" OR "emotion*" OR "psycholog*" OR "physiolog*" OR "recovery" OR “fatigue”) |

**Appendix 2. Classification strategy for studies based on design, context, participants, nature type, and health outcomes**

| Factor | Explanation |
| --- | --- |
| Study designs | Study designs were categorised as either interventional or non-interventional, depending on whether the study deliberately manipulated exposure to visible nature views from windows to assess health impacts. |
| Study context | Study contexts were classified into six categories based on the type of building and sample population: residential, workplace, educational, healthcare, leisure, and correctional. Hospital studies were categorized as either workplace or healthcare, depending on the sample population—studies focusing on hospital staff were classified under workplace, while those involving patients were classified under healthcare. |
| Type of participants | Participants were categorised into female-biased or non-female-biased samples. Female-biased samples were defined as those with more than 75% female participants. |
| Type of nature viewed | Nature types were classified into three categories: wild, managed, and general. Following Gaston and Soga (2020), wild nature includes organisms and environments that are minimally managed and close to pristine or wilderness conditions. Managed nature refers to environments highly influenced by human activity, such as urban greenspaces, gardens, or roadside trees. |
| Health outcomes | Health outcomes were grouped into three categories: physiological, psychological, and physical. The classification of length of hospitalisation depended on the underlying condition—if hospitalisation was due to a physical condition such as surgery, it was classified as a physical outcome, whereas if it was due to a psychological condition such as an affective disorder, it was classified as a psychological outcome. Similarly, pain-related outcomes were classified based on the type of measurement scale used. If the scale focused on the physical aspects of pain, the study result was classified as physical, whereas if it focused on the psychological aspects, the result was classified as psychological. |

**Appendix 3. Summary of quality appraisal of studies using the Mixed Methods Appraisal Tool (MMAT). The MMAT begins with two initial screening questions applicable to all studies, followed by five questions specific to the study design. Category 2 questions were applied to randomised controlled trials (RCTs), assessing criteria such as randomisation, baseline comparability, data completeness, blinding, and adherence to the intervention. For non-RCT quantitative studies (Category 3), the criteria included representation of the target population, use of appropriate measures, data completeness, control of confounding factors, and adherence to the intervention. Note: Y: Yes (if it met quality criterion); N: No (if it did not meet quality criterion); CT: Cannot tell (if it did not mention relevant information).**

| Reference | All studies | |  | Quantitative randomised controlled trials | | | | |  | Quantitative non-randomised | | | | |
| --- | --- | --- | --- | --- | --- | --- | --- | --- | --- | --- | --- | --- | --- | --- |
|  | 1. Are there clear research questions? | 2. Do the collected data allow to address the research questions? |  | 1. Is randomisation appropriately performed? | 2. Are the groups comparable at baseline? | 3. Are there complete outcome data? | 4. Are outcome assessors blinded to the intervention provided? | 5. Did the participants adhere to the assigned intervention? |  | 1. Are the participants representative of the target population? | 2. Are measurements appropriate regarding both the outcome and intervention (or exposure)? | 3. Are there complete outcome data? | 4. Are the confounders accounted for in the design and analysis? | 5. During the study period, is the intervention administered (or exposure occurred) as intended? |
| Elsadek et al. 2020 | Y | Y |  | - | - | - | - | - |  | CT | Y | CT | Y | CT |
| Braçe et al. 2020 | Y | Y |  | - | - | - | - | - |  | Y | Y | CT | Y | Y |
| Pearson et al. 2023 | Y | Y |  | - | - | - | - | - |  | CT | Y | Y | Y | Y |
| Raanaas et al. 2011 | Y | Y |  | - | - | - | - | - |  | CT | Y | CT | Y | Y |
| Yen et al. 2024 | Y | Y |  | - | - | - | - | - |  | CT | Y | CT | Y | Y |
| Zhang et al. 2024 | Y | Y |  | - | - | - | - | - |  | CT | Y | CT | N | Y |
| Li et al. 2024 | Y | Y |  | - | - | - | - | - |  | CT | Y | N | Y | CT |
| Patwary et al. 2024 | Y | Y |  | - | - | - | - | - |  | CT | Y | CT | Y | Y |
| Zhang et al. 2023 | Y | Y |  | - | - | - | - | - |  | CT | Y | CT | Y | Y |
| Ziabari et al. 2024 | Y | Y |  | - | - | - | - | - |  | Y | Y | Y | Y | CT |
| Garrido-Cumbrera et al. 2022 | Y | Y |  | - | - | - | - | - |  | N | Y | CT | Y | CT |
| Bi et al. 2022 | Y | Y |  | - | - | - | - | - |  | Y | Y | Y | CT | Y |
| Shentova et al. 2022 | Y | Y |  | - | - | - | - | - |  | CT | Y | CT | Y | CT |
| Li et al. 2021 | Y | Y |  | - | - | - | - | - |  | CT | Y | Y | CT | Y |
| Asim et al. 2021 | Y | Y |  | - | - | - | - | - |  | Y | Y | Y | CT | Y |
| Ribeiro et al. 2021 | Y | Y |  | - | - | - | - | - |  | CT | Y | CT | Y | CT |
| Spano et al. 2021 | Y | Y |  | - | - | - | - | - |  | CT | N | CT | Y | Y |
| Pouso et al. 2021 | Y | Y |  | - | - | - | - | - |  | CT | Y | CT | N | Y |
| Soga et al. 2021 | Y | Y |  | - | - | - | - | - |  | CT | Y | CT | Y | CT |
| Chang et al. 2020 | Y | Y |  | - | - | - | - | - |  | Y | Y | Y | Y | CT |
| Wang et al. 2019 | Y | Y |  | - | - | - | - | - |  | CT | Y | CT | Y | Y |
| van Esch et al. 2019 | Y | Y |  | - | - | - | - | - |  | Y | Y | Y | CT | Y |
| Korpela et al. 2017 | Y | Y |  | - | - | - | - | - |  | CT | Y | N | Y | CT |
| Ulrich 1984 | Y | Y |  | - | - | - | - | - |  | CT | Y | CT | N | Y |
| Foster et al. 2024 | Y | Y |  | - | - | - | - | - |  | CT | Y | Y | Y | CT |
| Mascherek et al. 2022 | Y | Y |  | - | - | - | - | - |  | CT | Y | CT | Y | Y |
| Emami et al. 2018 | Y | Y |  | - | - | - | - | - |  | N | Y | CT | N | CT |
| Li and Sullivan 2016 | Y | Y |  | Y | Y | CT | CT | CT |  | - | - | - | - | - |

**References**

Asim, F., Chani, P. S., & Shree, V. 2021. Impact of COVID-19 containment zone built-environments on students’ mental health and their coping mechanisms. Building and Environment 203: 108107.

Bi, W., Jiang, X., Li, H., Cheng, Y., Jia, X., Mao, Y., & Zhao, B. 2022. The more natural the window, the healthier the isolated people—A pathway analysis in Xi’an, China, during the COVID-19 pandemic. International Journal of Environmental Research and Public Health 19: 10165.

Braçe, O., Garrido-Cumbrera, M., Foley, R., Correa-Fernández, J., Suárez-Cáceres, G., & Lafortezza, R. 2020. Is a view of green spaces from home associated with a lower risk of anxiety and depression? International Journal of Environmental Research and Public Health 17: 7014.

Chang, C. C., Oh, R. R. Y., Le Nghiem, T. P., Zhang, Y., Tan, C. L., Lin, B. B., ... & Carrasco, L. R. 2020. Life satisfaction linked to the diversity of nature experiences and nature views from the window. Landscape and Urban Planning 202: 103874.

Emami, E., Amini, R., & Motalebi, G. 2018. The effect of nature as positive distractibility on the healing process of patients with cancer in therapeutic settings. Complementary Therapies in Clinical Practice 32: 70–73.

Elsadek, M., Liu, B., & Xie, J. 2020. Window view and relaxation: Viewing green space from a high-rise estate improves urban dwellers’ wellbeing. Urban Forestry & Urban Greening 55: 126846.

Foster, S., Kleeman, A., & Maitland, C. 2024. Research note: View from the top: Apartment residents’ views of nature and mental wellbeing during lockdown. Landscape and Urban Planning 247: 105072.

Garrido-Cumbrera, M., Foley, R., Correa-Fernández, J., Gonzalez-Marin, A., Braçe, O., & Hewlett, D. 2022. The importance for wellbeing of having views of nature from and in the home during the COVID-19 pandemic. Results from the GreenCOVID study. Journal of Environmental Psychology 83: 101864.

Korpela, K., De Bloom, J., Sianoja, M., Pasanen, T., & Kinnunen, U. 2017. Nature at home and at work: Naturally good? Links between window views, indoor plants, outdoor activities and employee well-being over one year. Landscape and Urban Planning 160: 38–47.

Li, D., & Sullivan, W. C. 2016. Impact of views to school landscapes on recovery from stress and mental fatigue. Landscape and Urban Planning 148: 149–158.

Li, H., Browning, M. H., Bardhan, M., Ying, M., Zhang, X., Cao, Y., & Zhang, G. 2024. Nature connectedness connects the visibility of trees through windows and mental wellbeing: A study on the “3 visible trees” component of the 3-30-300 rule. International Journal of Environmental Health Research: 1–13.

Li, H., Zhang, X., You, C., Chen, X., Cao, Y., & Zhang, G. 2021. Can viewing nature through windows improve isolated living? A pathway analysis on Chinese male prisoners during the COVID-19 epidemic. Frontiers in Psychiatry 12: 720722.

Mascherek, A., Weber, S., Riebandt, K., Cassanello, C., Leicht, G., Brick, T., ... & Kühn, S. 2022. On the relation between a green and bright window view and length of hospital stay in affective disorders. European Psychiatry 65: e21.

Patwary, M. M., Bardhan, M., İnan, H. E., Browning, M. H., Disha, A. S., Haque, M. Z., ... & Rodriguez-Morales, A. J. 2024. Exposure to urban green spaces and mental health during the COVID-19 pandemic: Evidence from two low and lower-middle-income countries. Frontiers in Public Health 12: 1334425.

Pearson, A. L., Brown, C. D., Reuben, A., Nicholls, N., Pfeiffer, K. A., & Clevenger, K. A. 2023. Elementary classroom views of nature are associated with lower child externalizing behavior problems. International Journal of Environmental Research and Public Health 20: 5653.

Pouso, S., Borja, Á., Fleming, L. E., Gómez-Baggethun, E., White, M. P., & Uyarra, M. C. 2021. Contact with blue-green spaces during the COVID-19 pandemic lockdown beneficial for mental health. Science of the Total Environment 756: 143984.

Raanaas, R. K., Patil, G. G., & Hartig, T. 2012. Health benefits of a view of nature through the window: A quasi-experimental study of patients in a residential rehabilitation center. Clinical Rehabilitation 26: 21–32.

Ribeiro, A. I., Triguero-Mas, M., Santos, C. J., Gómez-Nieto, A., Cole, H., Anguelovski, I., ... & Baró, F. 2021. Exposure to nature and mental health outcomes during COVID-19 lockdown. A comparison between Portugal and Spain. Environment International 154: 106664.

Shentova, R., De Vries, S., & Verboom, J. 2022. Well-being in the time of corona: Associations of nearby greenery with mental well-being during COVID-19 in The Netherlands. Sustainability 14: 10256.

Soga, M., Evans, M. J., Tsuchiya, K., & Fukano, Y. 2021. A room with a green view: The importance of nearby nature for mental health during the COVID-19 pandemic. Ecological Applications 31: e2248.

Spano, G., D’Este, M., Giannico, V., Elia, M., Cassibba, R., Lafortezza, R., & Sanesi, G. 2021. Association between indoor-outdoor green features and psychological health during the COVID-19 lockdown in Italy: A cross-sectional nationwide study. Urban Forestry & Urban Greening 62: 127156.

Ulrich, R. S. 1984. View through a window may influence recovery from surgery. Science 224: 420–421.

van Esch, E., Minjock, R., Colarelli, S. M., & Hirsch, S. 2019. Office window views: View features trump nature in predicting employee well-being. Journal of Environmental Psychology 64: 56–64.

Wang, C. H., Kuo, N. W., & Anthony, K. 2019. Impact of window views on recovery—An example of post-cesarean section women. International Journal for Quality in Health Care 31: 798–803.

Yen, M. H., Li, D., Weng, P. Y., & Chiang, Y. C. 2024. Impact of natural views on mental health during COVID-19 quarantine: A natural experiment. Journal of Environmental Psychology 100: 102455.

Zhang, Y. O., Tang, Y., Wang, X., & Tan, Y. 2024. The Effects of Natural Window Views in Classrooms on College Students’ Mood and Learning Efficiency. Buildings 14: 1557.

Zhang, Z., Zhang, H., Yang, H., & Zhong, B. 2023. Home Greenery: Alleviating Anxiety during Lockdowns with Varied Landscape Preferences. Sustainability 15: 15371.

Ziabari, S. M. Z., Andalib, E., Faghani, M., Roodsari, N. N., Arzhangi, N., Khesht-Masjedi, M. F., & Leyli, E. K. 2023. Evidence-based design in the hospital environment: A staff’s burnout study in the COVID-19 era. HERD: Health Environments Research & Design Journal 16: 236–249.

**Appendix 4. List of 104 study results from 28 case studies included in the meta-analysis.**

| Author | Year | Country | Context | Design | Nature type | Sample type | N | Gender distribution (female >75%) | Health outcome | Health outcome (type) | Effect size |
| --- | --- | --- | --- | --- | --- | --- | --- | --- | --- | --- | --- |
| Asim et al. | 2021 | India | Residential | Non-interventional | Managed | Students | 432 | Non-female-biased | Depression | Psychological | 0.51 |
| Asim et al. | 2021 | India | Residential | Non-interventional | Managed | Students | 432 | Non-female-biased | Anxiety | Psychological | 0.53 |
| Bi et al. | 2022 | China | Residential | Non-interventional | Managed | General | 508 | Non-female-biased | Life satisfaction | Psychological | 0.21 |
| Bi et al. | 2022 | China | Residential | Non-interventional | Managed | General | 508 | Non-female-biased | Loneliness | Psychological | 0.13 |
| Bi et al. | 2022 | China | Residential | Non-interventional | Managed | General | 508 | Non-female-biased | Being away | Psychological | 0.35 |
| Bi et al. | 2022 | China | Residential | Non-interventional | Managed | General | 508 | Non-female-biased | Compatibility | Psychological | 0.35 |
| Bi et al. | 2022 | China | Residential | Non-interventional | Managed | General | 508 | Non-female-biased | Anxiety | Psychological | 0.05 |
| Bi et al. | 2022 | China | Residential | Non-interventional | Managed | General | 508 | Non-female-biased | Depression | Psychological | 0.15 |
| Braçe et al. | 2020 | Spain | Residential | Non-interventional | Managed | General | 451 | Non-female-biased | Anxiety | Psychological | 0.26 |
| Braçe et al. | 2020 | Spain | Residential | Non-interventional | Managed | General | 464 | Non-female-biased | Depression | Psychological | 0.44 |
| Chang et al. | 2020 | Singapore | Residential | Non-interventional | General | General | 1262 | Non-female-biased | Life satisfaction | Psychological | 0.11 |
| Chang et al. | 2020 | Singapore | Workplace | Non-interventional | General | General | 1262 | Non-female-biased | Life satisfaction | Psychological | 0.06 |
| Elsadek et al. | 2020 | China | Workplace | Interventional | Managed | Workers | 30 | Female-biased | Brain activity | Physiological | 0.58 |
| Elsadek et al. | 2020 | China | Workplace | Interventional | Managed | Workers | 30 | Female-biased | Brain activity | Physiological | 0.88 |
| Elsadek et al. | 2020 | China | Workplace | Interventional | Managed | Workers | 30 | Female-biased | Brain activity | Physiological | 0.69 |
| Elsadek et al. | 2020 | China | Workplace | Interventional | Managed | Workers | 30 | Female-biased | Brain activity | Physiological | 0.81 |
| Elsadek et al. | 2020 | China | Workplace | Interventional | Managed | Workers | 30 | Female-biased | Heart rate | Physiological | 0.41 |
| Elsadek et al. | 2020 | China | Workplace | Interventional | Managed | Workers | 30 | Female-biased | Skin conductance | Physiological | 0.60 |
| Elsadek et al. | 2020 | China | Workplace | Interventional | Managed | Workers | 30 | Female-biased | Mood disturbance | Psychological | 0.84 |
| Emami et al. | 2018 | Iran | Healthcare | Non-interventional | General | Patients | 80 | Non-female-biased | Pain | Psychological | 0.38 |
| Emami et al. | 2018 | Iran | Healthcare | Non-interventional | General | Patients | 80 | Non-female-biased | Anxiety | Psychological | 0.65 |
| Emami et al. | 2018 | Iran | Healthcare | Non-interventional | General | Patients | 80 | Non-female-biased | Anxiety | Psychological | 0.67 |
| Foster et al. | 2024 | Australia | Residential | Non-interventional | General | General | 192 | Non-female-biased | Wellbeing | Psychological | 0.18 |
| Garrido-Cumbrera et al. | 2022 | Spain/ Ireland/ UK | Residential | Non-interventional | General | General | 3109 | Non-female-biased | Poor wellbeing | Psychological | 0.01 |
| Garrido-Cumbrera et al. | 2022 | Spain/ Ireland/ UK | Residential | Non-interventional | General | General | 3109 | Non-female-biased | Depression | Psychological | 0.00 |
| Korpela et al. | 2017 | Finland | Workplace | Non-interventional | Managed | Workers | 841 | Non-female-biased | Creativity | Psychological | 0.03 |
| Korpela et al. | 2017 | Finland | Residential | Non-interventional | Managed | Workers | 841 | Non-female-biased | Creativity | Psychological | 0.15 |
| Korpela et al. | 2017 | Finland | Workplace | Non-interventional | Managed | Workers | 841 | Non-female-biased | Vigor | Psychological | 0.05 |
| Korpela et al. | 2017 | Finland | Residential | Non-interventional | Managed | Workers | 841 | Non-female-biased | Vigor | Psychological | 0.15 |
| Korpela et al. | 2017 | Finland | Workplace | Non-interventional | Managed | Workers | 841 | Non-female-biased | Happiness | Psychological | 0.06 |
| Korpela et al. | 2017 | Finland | Residential | Non-interventional | Managed | Workers | 841 | Non-female-biased | Happiness | Psychological | 0.12 |
| Korpela et al. | 2017 | Finland | Workplace | Non-interventional | Managed | Workers | 841 | Non-female-biased | Vitality | Psychological | 0.09 |
| Korpela et al. | 2017 | Finland | Residential | Non-interventional | Managed | Workers | 841 | Non-female-biased | Vitality | Psychological | 0.12 |
| Li and Sullivan | 2016 | US | Educational | Non-interventional | Managed | Students | 94 | Non-female-biased | Attention | Psychological | 0.36 |
| Li and Sullivan | 2016 | US | Educational | Non-interventional | Managed | Students | 94 | Non-female-biased | Stress | Psychological | 0.25 |
| Li et al. | 2024 | China | Residential | Non-interventional | Managed | Students | 618 | Female-biased | General mental health | Psychological | 0.14 |
| Li et al. | 2021 | China | Correctional | Non-interventional | Managed | Prisoners | 269 | Non-female-biased | Wellbeing | Psychological | 0.19 |
| Li et al. | 2021 | China | Correctional | Non-interventional | Managed | Prisoners | 269 | Non-female-biased | Life satisfaction | Psychological | 0.15 |
| Li et al. | 2021 | China | Correctional | Non-interventional | Managed | Prisoners | 269 | Non-female-biased | Destress tolerance | Psychological | 0.18 |
| Li et al. | 2021 | China | Correctional | Non-interventional | Managed | Prisoners | 269 | Non-female-biased | Loneliness | Psychological | 0.15 |
| Li et al. | 2021 | China | Correctional | Non-interventional | Managed | Prisoners | 269 | Non-female-biased | Anxiety | Psychological | 0.15 |
| Li et al. | 2021 | China | Correctional | Non-interventional | Managed | Prisoners | 269 | Non-female-biased | Depression | Psychological | 0.16 |
| Mascherek et al. | 2022 | Germany | Healthcare | Non-interventional | Managed | Patients | 244 | Non-female-biased | Length of hospitalisation | Psychological | 0.13 |
| Patwary et al. | 2024 | Bangladesh | Residential | Non-interventional | General | General | 556 | Non-female-biased | Anxiety | Psychological | 0.06 |
| Patwary et al. | 2024 | Egypt | Residential | Non-interventional | General | General | 660 | Non-female-biased | Anxiety | Psychological | -0.04 |
| Patwary et al. | 2024 | Bangladesh | Residential | Non-interventional | General | General | 556 | Non-female-biased | Depression | Psychological | 0.07 |
| Patwary et al. | 2024 | Egypt | Residential | Non-interventional | General | General | 660 | Non-female-biased | Depression | Psychological | 0.12 |
| Pearson et al. | 2023 | US | Educational | Non-interventional | Managed | Students | 86 | Female-biased | Externalising behaviour | Psychological | 0.63 |
| Pearson et al. | 2023 | US | Educational | Non-interventional | Managed | Students | 86 | Female-biased | Attention problems | Psychological | -0.05 |
| Pouso et al. | 2021 | Spain | Residential | Non-interventional | General | General | 3403 | Non-female-biased | Depression | Psychological | 0.03 |
| Pouso et al. | 2021 | Spain | Residential | Non-interventional | General | General | 3403 | Non-female-biased | Anxiety | Psychological | 0.02 |
| Raanaas et al. | 2011 | Norway | Residential | Interventional | Wild | Patients | 278 | Non-female-biased | Self-perceived physical health | Physical | 0.10 |
| Raanaas et al. | 2011 | Norway | Residential | Interventional | Wild | Patients | 278 | Non-female-biased | Subjective wellbeing | Psychological | 0.17 |
| Ribeiro et al. | 2021 | Portugal | Residential | Non-interventional | General | General | 1638 | Female-biased | Psychological distress | Psychological | 0.05 |
| Ribeiro et al. | 2021 | Portugal | Residential | Non-interventional | General | General | 1638 | Female-biased | Somatisation | Psychological | 0.06 |
| Ribeiro et al. | 2021 | Portugal | Residential | Non-interventional | General | General | 1638 | Female-biased | Stress | Psychological | 0.09 |
| Ribeiro et al. | 2021 | Spain | Residential | Non-interventional | General | General | 1519 | Non-female-biased | Psychological distress | Psychological | -0.03 |
| Ribeiro et al. | 2021 | Spain | Residential | Non-interventional | General | General | 1519 | Non-female-biased | Somatisation | Psychological | 0.02 |
| Ribeiro et al. | 2021 | Spain | Residential | Non-interventional | General | General | 1519 | Non-female-biased | Stress | Psychological | -0.03 |
| Shentova et al. | 2022 | Netherlands | Residential | Non-interventional | Managed | General | 521 | Non-female-biased | General mental health | Psychological | 0.18 |
| Soga et al. | 2021 | Japan | Residential | Non-interventional | Managed | General | 3000 | Non-female-biased | Self-esteem | Psychological | 0.05 |
| Soga et al. | 2021 | Japan | Residential | Non-interventional | Managed | General | 3000 | Non-female-biased | Life satisfaction | Psychological | 0.09 |
| Soga et al. | 2021 | Japan | Residential | Non-interventional | Managed | General | 3000 | Non-female-biased | Happiness | Psychological | 0.07 |
| Soga et al. | 2021 | Japan | Residential | Non-interventional | Managed | General | 3000 | Non-female-biased | Loneliness | Psychological | 0.04 |
| Soga et al. | 2021 | Japan | Residential | Non-interventional | Managed | General | 3000 | Non-female-biased | Depression/anxiety | Psychological | 0.04 |
| Spano et al. | 2021 | Italy | Residential | Non-interventional | General | General | 3886 | Non-female-biased | Anxiety | Psychological | 0.22 |
| Spano et al. | 2021 | Italy | Residential | Non-interventional | General | General | 3886 | Non-female-biased | Anger | Psychological | 0.28 |
| Spano et al. | 2021 | Italy | Residential | Non-interventional | General | General | 3886 | Non-female-biased | Fear | Psychological | 0.26 |
| Spano et al. | 2021 | Italy | Residential | Non-interventional | General | General | 3886 | Non-female-biased | Confusion | Psychological | 0.24 |
| Spano et al. | 2021 | Italy | Residential | Non-interventional | General | General | 3886 | Non-female-biased | Moodiness | Psychological | 0.26 |
| Spano et al. | 2021 | Italy | Residential | Non-interventional | General | General | 3886 | Non-female-biased | Boredom | Psychological | 0.35 |
| Spano et al. | 2021 | Italy | Residential | Non-interventional | General | General | 3886 | Non-female-biased | Irritability | Psychological | 0.30 |
| Spano et al. | 2021 | Italy | Residential | Non-interventional | General | General | 3886 | Non-female-biased | Recurrent thoughts/dreams | Psychological | 0.20 |
| Spano et al. | 2021 | Italy | Residential | Non-interventional | General | General | 3886 | Non-female-biased | Poor concentration | Psychological | 0.24 |
| Spano et al. | 2021 | Italy | Residential | Non-interventional | General | General | 3886 | Non-female-biased | Sleep disturbance | Psychological | 0.22 |
| Ulrich | 1984 | US | Healthcare | Interventional | Managed | Patients | 36 | Non-female-biased | Length of hospitalisation | Physical | 0.46 |
| Ulrich | 1984 | US | Healthcare | Interventional | Managed | Patients | 44 | Non-female-biased | Mental health | Psychological | 0.74 |
| van Esch et al. | 2019 | US | Workplace | Non-interventional | General | Workers | 303 | Non-female-biased | Emotional exhaustion | Psychological | 0.19 |
| van Esch et al. | 2019 | US | Workplace | Non-interventional | General | Workers | 303 | Non-female-biased | Apprehension | Psychological | 0.27 |
| van Esch et al. | 2019 | US | Workplace | Non-interventional | General | Workers | 303 | Non-female-biased | Restoration | Psychological | 0.48 |
| van Esch et al. | 2019 | US | Workplace | Non-interventional | General | Workers | 303 | Non-female-biased | Physical wellbeing | Physical | 0.01 |
| Wang et al. | 2019 | Taiwan | Healthcare | Interventional | Managed | Patients | 296 | Female-biased | Analgesic prescription | Physical | -0.06 |
| Wang et al. | 2019 | Taiwan | Healthcare | Interventional | Managed | Patients | 296 | Female-biased | Pain experience | Physical | 0.00 |
| Wang et al. | 2019 | Taiwan | Healthcare | Interventional | Managed | Patients | 296 | Female-biased | Pain severity | Physical | -0.04 |
| Wang et al. | 2019 | Taiwan | Healthcare | Interventional | Managed | Patients | 296 | Female-biased | Impact of pain on life and mood | Psychological | 0.02 |
| Yen et al. | 2024 | Taiwan | Leisure | Non-interventional | General | General | 81 | Female-biased | Positive mental health | Psychological | 0.27 |
| Yen et al. | 2024 | Taiwan | Leisure | Non-interventional | General | General | 81 | Female-biased | Depression | Psychological | 0.37 |
| Yen et al. | 2024 | Taiwan | Leisure | Non-interventional | General | General | 81 | Female-biased | Stress | Psychological | 0.19 |
| Yen et al. | 2024 | Taiwan | Leisure | Non-interventional | General | General | 81 | Female-biased | Positive mental health | Psychological | 0.29 |
| Yen et al. | 2024 | Taiwan | Leisure | Non-interventional | General | General | 81 | Female-biased | Depression | Psychological | 0.58 |
| Yen et al. | 2024 | Taiwan | Leisure | Non-interventional | General | General | 81 | Female-biased | Stress | Psychological | 0.34 |
| Zhang et al. | 2024 | China | Educational | Interventional | Managed | Students | 30 | Non-female-biased | Brain activity | Physiological | 0.46 |
| Zhang et al. | 2024 | China | Educational | Interventional | Managed | Students | 30 | Non-female-biased | Brain activity | Physiological | 0.48 |
| Zhang et al. | 2024 | China | Educational | Interventional | Managed | Students | 30 | Non-female-biased | Brain activity | Physiological | 0.50 |
| Zhang et al. | 2024 | China | Educational | Interventional | Managed | Students | 30 | Non-female-biased | Brain activity | Physiological | 0.62 |
| Zhang et al. | 2024 | China | Educational | Interventional | Managed | Students | 30 | Non-female-biased | Brain activity | Physiological | 0.19 |
| Zhang et al. | 2024 | China | Educational | Interventional | Managed | Students | 30 | Non-female-biased | Attention | Psychological | 0.24 |
| Zhang et al. | 2024 | China | Educational | Interventional | Managed | Students | 30 | Non-female-biased | Heart rate | Physiological | 0.17 |
| Zhang et al. | 2024 | China | Educational | Interventional | Managed | Students | 30 | Non-female-biased | Attention task | Psychological | 0.45 |
| Zhang et al. | 2024 | China | Educational | Interventional | Managed | Students | 30 | Non-female-biased | Mood disturbance | Psychological | 0.85 |
| Zhang et al. | 2023 | China | Residential | Non-interventional | Managed | General | 1287 | Non-female-biased | Depression | Psychological | 0.07 |
| Ziabari et al. | 2024 | Iran | Workplace | Non-interventional | Managed | Workers | 406 | Female-biased | Emotional exhaustion | Psychological | 0.39 |
| Ziabari et al. | 2024 | Iran | Workplace | Non-interventional | Managed | Workers | 406 | Female-biased | Depersonalisation | Psychological | 0.34 |
| Ziabari et al. | 2024 | Iran | Workplace | Non-interventional | Managed | Workers | 406 | Female-biased | Reduced personal accomplishment | Psychological | 0.39 |

**Appendix 5. Formulae for converting effect sizes to Pearson’s correlation (*r*).**

| Statistics to be converted | Formula for transformation to *r* | Comment |
| --- | --- | --- |
| Spearman’s *r* (*r_s_*) | *r* = 2 * sin(π / 6 * *r_s_*) |  |
| Standardized regression coefficients (*β*) | *r* = *β* + 0.05 * λ | λ is a constant that takes the value of 1 if β ≥ 0, and 0 if β < 0. |
| Partial eta squared (*η²*) | r = √*η²* |  |
| Chi-squared (*x^2^*) | *r* = √(*χ²* / (*χ²* + *df*)) |  |
| Univariate odds ratios (*ORs*) | *r* = (√*OR* - 1) / (√*OR* + 1) |  |
| Coefficient of determination (*R^2^*) | *r* = √(*R²*) |  |
| *z* scores (*z*) | *r* = *z* / √*n* |  |
| *t* value (*t*) | *r* = √(*t²* / (*t²* + *df*)) | If the *t*-statistic was not reported, we approximated it using the regression coefficient and the standard errors. For t-tests, where the means and standard deviations of the two groups were available, we calculated the *t*-statistic based on these values, following the standard formula for the independent t-test. |

**Appendix 6. Histogram of the standardised mean correlation coefficient (*r*) calculated using bootstrap resampling with 1,000 resamples. See the main text for additional context.**


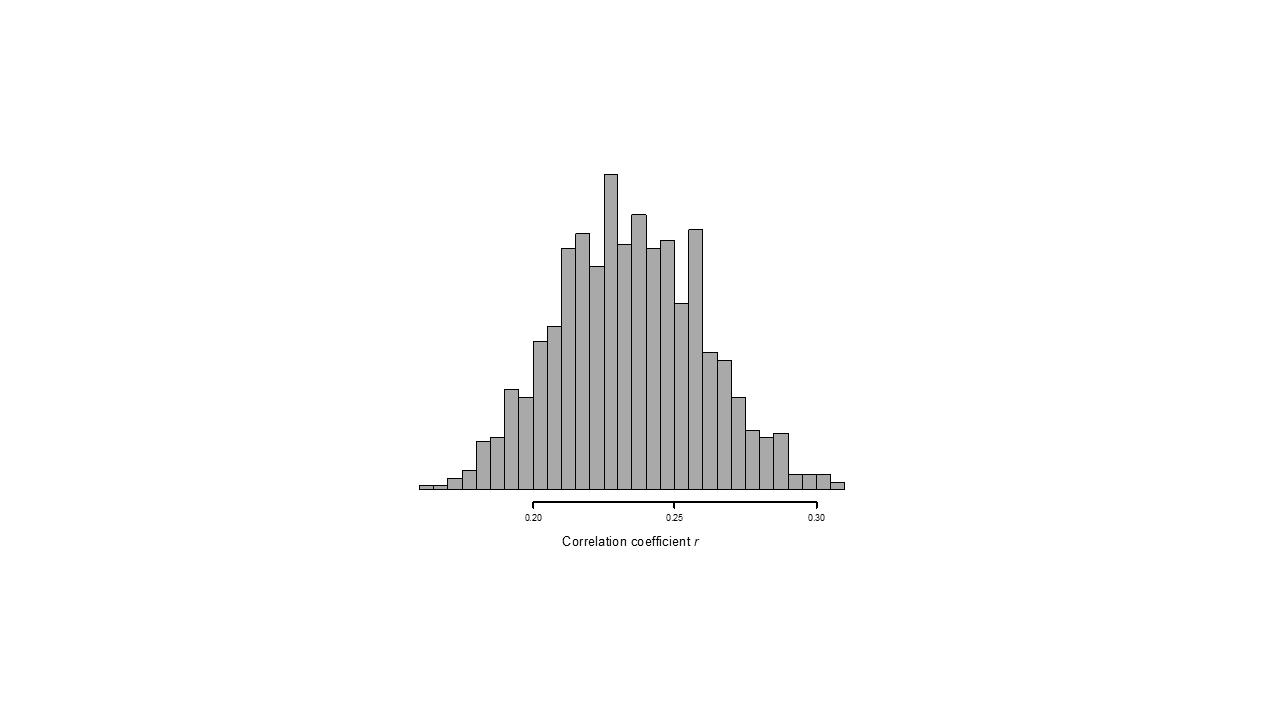

Supplement: biaf089_Supplemental_File [file biaf089_supplemental_file.docx]
